# Effect of manual reduction and indirect decompression on thoracolumbar burst fracture: a comparison study

**DOI:** 10.1186/s13018-020-02075-w

**Published:** 2020-11-13

**Authors:** Jian Huang, Limin Zhou, Zhaodong Yan, Zongbo Zhou, Xuejian Gou

**Affiliations:** Department of Orthopaedic, Haikou Hospital of Traditional Chinese Medicine, 2 Poxiang Road, Longhua District, Haikou, Hainan Province People’s Republic of China

**Keywords:** Manipulative reduction, Indirect decompression, Thoracolumbar burst fractures

## Abstract

**Objective:**

To evaluate the effect of manual reduction and indirect decompression on thoracolumbar burst fracture.

**Methods:**

Sixty patients with thoracolumbar burst fracture who were hospitalized from January 2018 to October 2019 were selected and divided into an experimental group (33 cases) and control group (27 cases) according to different treatment methods. The experimental group was treated with manual reduction and indirect decompression, while the control group was not treated with manual reduction. The operation time and intraoperative blood loss were recorded. VAS score was used to evaluate the improvement of pain. The anterior height of the injured vertebra, wedge angle of the injured vertebral body, and encroachment ratio of the injured vertebral canal were used to evaluate the spinal canal decompression and fracture reduction. JOA score was used to evaluate the improvement of spinal function.

**Results:**

There was no significant difference in operation time and intraoperative blood loss between the two groups. Compared with the control group, the VAS score and the wedge angle of the injured vertebral body of the experimental group 3 days after the operation and the last follow-up were significantly lower than that of the control group, and the difference was statistically significant. The ratio of the anterior height of the injured vertebra of the experimental group 3 days after the operation and the last follow-up was significantly higher than that of the control group, and the difference was statistically significant. The difference of the encroachment ratio of the injured vertebral canal between preoperation and 3 days after operation was significantly higher than that of the control group, and the difference was statistically significant. The bladder function of JOA 3 days after the operation of the experimental group was significantly higher than that of the control group, and the difference was statistically significant. And the rest aspect of JOA on 3 days after the operation and last follow-up of the experimental group has no significant difference compared with the control group.

**Conclusion:**

Manipulative reduction and indirect decompression can obtain a better clinical effect in the treatment of thoracolumbar burst fractures.

## Background

Thoracolumbar burst fracture most often occurs in the thoracolumbar segment (T11-L2) [1]. The thoracolumbar segment is located between the stable kyphosis thoracic vertebrae and the flexible lordosis lumbar vertebrae. It is the intersection point of the thoracic and lumbar vertebrae and also the stress concentration point [2]. Therefore, when the compression force is given, it is easy to cause a vertebral fracture. When the compression force is enough, the vertebral body will break radially, which will cause a burst fracture. Thoracolumbar segment burst fracture is often seen in falling injury and traffic accident injury. Denis put forward the three-column theory and divided the fracture into compression type, burst type, flexion stretch type, and fracture-dislocation type [3]; burst fracture type was further divided into 5 subtypes a–e by Denis. The most common clinical type is type B [4], which refers to the burst fracture involving only the upper endplate. Due to the loss of vertebral height, part of the posterior wall of the vertebral body protrudes into the spinal canal, resulting in kyphosis. Therefore, the burst fractures are unstable fractures [5]. Because the posterior edge of the vertebral body protrudes into the spinal canal, burst fracture is easy to cause symptoms of the spinal cord or nerve compression. At present, most scholars at home and abroad believe that even if there is no symptom of the spinal cord or nerve compression, surgical treatment is still advocated [6]. The purpose of surgical treatment is to restore the stability of the spine and to decompress the nerve structure in patients with progressive aggravation of the spinal cord or nerve compression symptoms [7]. Some studies have shown that the bone fragments protruding into the spinal canal can be absorbed by themselves, and if the spine is kept in order, there will be no secondary spinal stenosis in the later stage. Therefore, even if the spinal canal occupies more than 50% and there are no neurological damage symptoms, decompression is not required. The compression methods include direct decompression and indirect decompression. In this study, manual reduction and indirect decompression technique were used to treat thoracolumbar burst fracture, using X-ray and CT scan data to create anatomical models of human anatomy, to evaluate the surgical effect, which was also in line with the concept of translational orthopedics. Mediouni proposed a “T-Model,” through the participation of a multidisciplinary team, and basic research scientists participated in the operation of the clinical surgeon, aiming to bridge the gap between basic science and clinical science and improve the surgeon’s surgical competencies [8–10].

## Materials and methods

### Patient selection method

Inclusion criteria include the following: (a) there was a clear history of trauma; (b) chest and waist pain, limited movement; (c) Denis type B thoracolumbar burst fracture diagnosed by CT scan [11]; (d) TLICS score is 4 points or greater [12, 13]; (e) Asia Grade E; (f) 18 years and older; (7) follow-up time is 1 year or longer; and (g) institutional Review Board approval was obtained before the study commenced.

Exclusion criteria include the following: (a) the patients were treated with the operation for more than 72 h, (b) with fracture of other parts, (c) treated with manipulation after injury, (d) patients with nerve injury and progressive aggravation, and (e) patients with coagulation dysfunction.

### General information

Sixty patients with thoracolumbar burst fracture who were hospitalized from January 2018 to October 2019 were selected and divided into an experimental group (33 cases) and control group (27 cases) according to different treatment methods.

### Surgical technique

All operations were performed by the chief surgeon of spinal surgery. All patients were anesthetized with combined spinal and epidural anesthesia. All patients were placed in a prone position with pillows on their chest and ilium to make the anterior column of the spine under tension in order to reset the spine curvature. The pedicle of the fractured vertebral body was located and marked on the skin by C-arm fluoroscopy. The back median longitudinal incision was about 10 cm in length according to fractured vertebrae, and the skin and subcutaneous tissue were cut to the lumbodorsal fascia, and the skin was pulled to both sides of the spinous process. At 1.0–2.0 cm on both sides of the spinous process, the lumbodorsal fascia was incised longitudinally. In the space between the longissimus muscle and multifidus muscle, the index finger was used to blunt separate and touch the articular process joint and transverse process of the fractured vertebral body, the upper and lower vertebral body. After the attached muscles were cauterized and peeled off with an electric knife, the opening cone was used to open the pedicle cortex, and pushed forward and tilted inward slowly according to the hand feeling. The insertion of the marker and the fluoroscopy position of the marker was satisfactory. Six pedicle screws were inserted into the pedicle, the single-axis pedicle screws were inserted into the upper and lower normal vertebrae, and the universal axis pedicle screws were inserted into the fractured vertebral. The pedicle screw length of the fracture vertebral was selected to be slightly shorter than the upper and lower normal vertebrae by 5–10 mm. And then the prebent connecting rod was inserted. Firstly, the injured vertebral pedicle screw is tightened, then the lower normal vertebral pedicle screw was tightened, and finally, the upper normal vertebral pedicle screw was tightened.

**Fig. 1:**
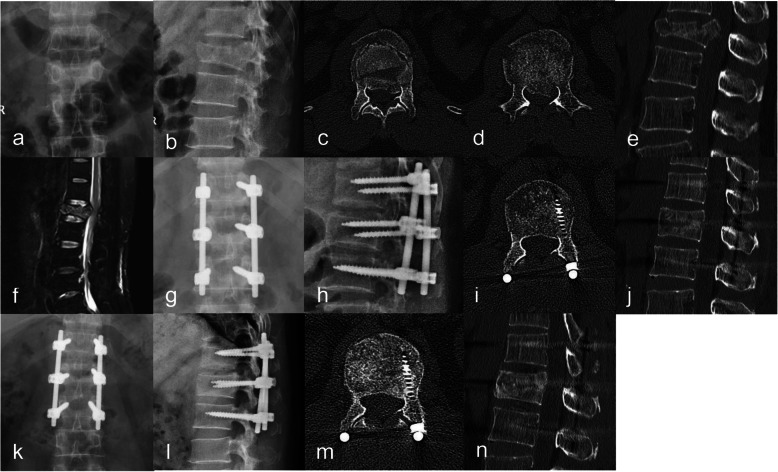
A 39-year-old man was diagnosed with an L1 vertebral burst fracture. **a** Preoperative anterior and posterior X-ray of the lumbar spine. **b** Preoperative lateral X-ray of the lumbar spine. **c** Preoperative CT axial position. **d** Preoperative CT axial position. **e** Preoperative CT sagittal position. **f** Preoperative MRI sagittal position. **g** Anterior and posterior X-ray of the lumbar spine on 3 days after the operation. **h** Lateral X-ray of the lumbar spine on 3 days after the operation. **i** CT axial position on 3 days after the operation. **j** CT sagittal position on 3 days after the operation. **k** Anterior and posterior X-ray of the lumbar spine on the last follow-up. l Lateral X-ray of the lumbar spine on the last follow-up. m CT axial position on the last follow-up. **n** CT sagittal position on the last follow-up

In the experimental group, before tightening the pedicle screw of the lower normal vertebral body, the assistant inserted the screwdriver into the screw cap. The operator placed the palm of the one hand on the spinous process, the longitudinal axis of the hand was parallel to the spinous process, and the palm of the other hand was placed on the back of the front hand, and the pressure was applied vertically to the ventral side in order to reset the fracture. The force was appropriate to feel the spinous process move to the ventral side. The procedure lasted 20 s. Another assistant should prop apart the injured vertebral pedicle screw and the normal vertebral pedicle screw. Then, the assistant tightened the screw cap. The injured vertebral and upper normal vertebral were treated with the same method (Fig. 1).

**Fig. 2:**
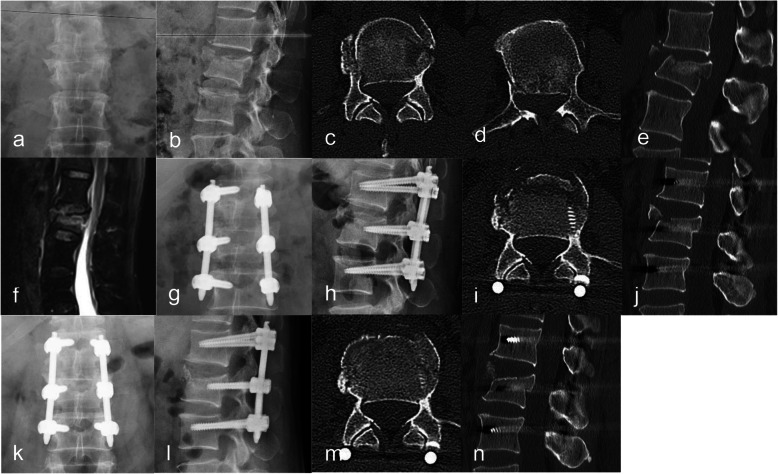
A 42-year-old man was diagnosed with an L2 vertebral burst fracture. **a** Preoperative anterior and posterior X-ray of the lumbar spine. **b** Preoperative lateral X-ray of the lumbar spine. **c** Preoperative CT axial position. **d** Preoperative CT axial position. **e** Preoperative CT sagittal position. **f** Preoperative MRI sagittal position. **g** Anterior and posterior X-ray of the lumbar spine on 3 days after the operation. **h** Lateral X-ray of the lumbar spine on 3 days after the operation. **i** CT axial position on 3 days after the operation. **j** CT sagittal position on 3 days after the operation. **k** Anterior and posterior X-ray of the lumbar spine on the last follow-up. **l** Lateral X-ray of the lumbar spine on the last follow-up. **m** CT axial position on the last follow-up. **n** CT sagittal position on the last follow-up

In the control group, there was no manual pressure reduction when the injured vertebral and the upper and lower normal vertebral were propped apart (Fig. 2).

### Postoperative managements

All patients were treated with antibiotics for 48 h after operation. They were treated with dehydration and neurotrophic therapy routinely. Three to 5 days after the operation, the patients should wear the thoracolumbar brace and try to walk.

### Efficacy evaluation

All patients were followed-up for at least 12 months after treatment. The operation time and intraoperative blood loss of all patients were recorded. Intraoperative blood loss = (preoperative hemoglobin - postoperative hemoglobin)/preoperative hemoglobin × 100%. VAS pain score standard was used to evaluate the improvement of pain. From 0 to 10 points, the higher the score, the more obvious the pain. VAS scores before the operation, 3 days after the operation, and the last follow-up were recorded. The anterior vertical height of the median sagittal plane of the vertebral body on the lateral X-ray film was measured. The ratio of the anterior height of injured vertebra = (anterior height of injured vertebra/average height of upper and lower vertebrae of the injured vertebra) × 100%. The anterior height of the injured vertebral body was recorded before the operation, 3 days after the operation, and the last follow-up. The angle between the extension line of upper and lower endplates of the median sagittal plane of the vertebral body on lateral X-ray film was measured. The wedge angle of the injured vertebral body was recorded before the operation, 3 days after the operation and the last follow-up. The encroachment ratio of the injured vertebral canal was calculated according to the axial image of the injured vertebra on a plain CT scan. The encroachment ratio = the maximum value of the bone cortex protruding into the spinal canal at the posterior edge of the vertebral body/sagittal diameter of the spinal canal × 100%. The encroachment ratio of the injured vertebral canal was recorded before and 3 days after the operation. JOA score was used to evaluate the improvement of spinal function. JOA score was evaluated from subjective symptoms, clinical signs, limitation of daily activities, and bladder function. From 0 to 29 points, the lower the score, the more obvious the dysfunction. JOA scores were recorded before the operation, 3 days after the operation, and the last follow-up.

### Statistical methods

SPSS 26.0 was used for data analysis. The measurement data were expressed by mean ± standard deviation. For intergroup comparison, variance homogeneity *F* test was used first, then independent sample *t/t'* test was used, and paired sample *t* test was used for intragroup comparison. The count data were expressed by the number of cases and percentage, and the comparison of counting data was performed by chi-squared test. Test level *α* = 0.05, bilateral test.

## Results

### General results

All patients had no serious complications, such as nerve root and spinal cord injury, screw and rod broken, hematoma, or wound infection. There was no significant difference in gender, age, injury time, and clinical manifestations between the two groups (Table 1).

**Table 1:** Patient characteristics

	Experimental group	Control group	*P*^▽^
Number	33	27	
Injury time (hr ± SD)	4.42 ± 1.26	4.29 ± 1.83	0.429
Gender (male to female)	28:5	25:2	0.759
Clinical manifestations			
Pain	33	27	0.282
Transient dysuria	22	19	0.296
Indwelling catheter for dysuria	12	11	0.973
Constipation	5	3	0.853
Bulbocavernous reflex was positive	33	27	0.282
Anal reflex was positive	33	27	0.282

^▽^*P* value of the injury time is calculated by independent sample *t* test (*F* = 0.721, *P* = 0.193 > 0.05); others are calculated by chi-squared test

*hr* hour, *SD* standard deviation

### Comparison of operation time, intraoperative blood loss, and VAS scores

There was no significant difference in operation time and intraoperative blood loss between the two groups. There was no significant difference in VAS score between the two groups on preoperation (*t* = 0.479, *P* > 0.05). In each group, there were significant differences in VAS score between the preoperation and 3 days after the operation, and last follow-up. Compared with the control group, the VAS score of the experimental group on 3 days after the operation and the last follow-up was significantly lower than that of the control group, and the difference was statistically significant (Table 2).

**Table 2:** Comparison of operation time, intraoperative blood loss, and VAS scores in two groups

	Experimental group	Control group	*t/t'*	*P*
Operation time (min)	62 ± 7.18	61 ± 6.21	0.019	1.713
Intraoperative blood loss (%)	7.1 ± 3.43	6.8 ± 3.10	0.285	0.934
VAS				
Preoperation	7.31 ± 1.51	7.02 ± 1.95	0.479	0.774
3 days after operation	2.23 ± 0.23* *t* = 5.331, *P* < 0.05	2.93 ± 0.42* *t* = 5.832, *P* < 0.05	3.693	0.036*
Last follow-up	0.21 ± 0.13* *t* = 6.520, *P* < 0.05	1.01 ± 0.97* *t* = 6.357, *P* < 0.05	3.511	0.025*

Values are mean ± SD

*Statistically significant

### Comparison of the ratio of the anterior height of the injured vertebra

There was no significant difference in the ratio of the anterior height of the injured vertebra between the two groups before the operation. In each group, there were significant differences in the ratio of the anterior height of injured vertebra between the preoperation and 3 days after the operation, and last follow-up. In the control group, there was a significant difference in the ratio of the anterior height of injured vertebra between 3 days after the operation and the last follow-up. But in the experimental group, there was no significant difference in the ratio of the anterior height of injured vertebra between 3 days after the operation and the last follow-up. Compared with the control group, the ratio of the anterior height of the injured vertebra of the experimental group 3 days after the operation and the last follow-up were significantly higher than that of the control group, and the difference was statistically significant (Table 3).

**Table 3:** Comparison of the ratio of the anterior height of the injured vertebra

	Experimental group	Control group	*t/t'*	*P*
Ratio of the anterior height of the injured vertebra				
Preoperation	50.95 ± 8.31	52.18 ± 5.92	0.713	0.687
3 days after operation	95.25 ± 4.82*	90.36 ± 2.39*	5.352	0.011*
	*t* = 5.891, *P* < 0.05	*t* = 5.421, *P* < 0.05		
Last follow-up	94.93 ± 5.13*	86.94 ± 3.90*	4.472	0.021*
	*t* = 3.702, *P* < 0.05	*t* = 4.051, *P* < 0.05		
	*t* = 0.681, *P* > 0.05^▽^	*t* = 3.823, *P* < 0.05^▽^		

Values are mean ± SD

*Statistically significant

^▽^Ratio of the anterior height of injured vertebra between 3 days after the operation and the last follow-up

### Comparison of a wedge angle of the injured vertebral body

There was no significant difference in the wedge angle of the injured vertebral body between the two groups before the operation. In each group, there were significant differences in the wedge angle of the injured vertebral body between the preoperation and 3 days after the operation, and last follow-up. There was no significant difference in the wedge angle of the injured vertebral body between 3 days after the operation and the last follow-up in two groups. Compared with the control group, the wedge angle of the injured vertebral body of the experimental group on 3 days after the operation, and the last follow-up were significantly lower than that of the control group, and the difference was statistically significant (Table 4).

**Table 4:** Comparison of the wedge angle of the injured vertebral body

	Experimental group	Control group	*t/t'*	*P*
Wedge angle of the injured vertebral body				
Preoperation	24.46 ± 3.42	23.94 ± 3.21	0.638	0.707
3 days after operation	1.21 ± 0.32*	4.93 ± 1.15*	5.725	0.010*
	*t* = 5.638, *P* < 0.05	*t* = 4.379, *P* < 0.05		
Last follow-up	1.15 ± 0.10*	5.01 ± 0.97*	4.625	0.019*
	*t* = 3.898, *P* < 0.05	*t* = 3.752, *P* < 0.05		
	*t* = 0.852, *P* > 0.05^▽^	*t* = 0.068, *P* > 0.05^▽^		

Values are mean ± SD

*Statistically significant

^▽^Wedge angle of the injured vertebral body between 3 days after the operation and the last follow-up

### Comparison of encroachment ratio of the injured vertebral canal

There was no significant difference in the encroachment ratio of the injured vertebral canal between the two groups before the operation. In each group, there was a significant difference in the encroachment ratio of the injured vertebral canal between the preoperation and 3 days after the operation. Compared with the control group, the difference of the encroachment ratio of the injured vertebral canal between preoperation and 3 days after the operation was significantly higher than that of the control group, and the difference was statistically significant (Table 5).

**Table 5:** Comparison of encroachment ratio of the injured vertebral canal

	Experimental group	Control group	*t/t'*	*P*
Encroachment ratio of the injured vertebral canal				
Preoperation	27.46 ± 8.73	28.94 ± 5.38	0.572	0.747
3 days after operation	1.63 ± 0.59*	6.52 ± 4.56*		
	*t* = 4.921, *P* < 0.05	*t* = 4.840, *P* < 0.05		
Difference of the encroachment ratio of the injured vertebral canal between the preoperation and 3 days after the operation	25.46 ± 4.26	21.51 ± 1.36	3.295	0.033*

Values are mean ± SD

*Statistically significant

### Comparison of JOA scores

There was no significant difference in the JOA scores between the two groups before the operation. In each group, there was a significant difference in the JOA scores between the preoperation and 3 days after the operation and last follow-up. Compared with the control group, the subjective symptoms, clinical signs, and daily activity limitation of JOA on 3 days after the operation and the subjective symptoms, clinical signs, daily activity limitation, and bladder function of JOA on the last follow-up of the experimental group were no significant difference. But the bladder function of JOA 3 days after the operation of the experimental group was significantly higher than that of the control group, and the difference was statistically significant (Table 6).

**Table 6:** Comparison of JOA scores

JOA scores	Experimental group	Control group	*t/t'*	*P*
Subjective symptoms				
Preoperation	0.69 ± 0.05	0.71 ± 0.02	0.472	0.846
3 days after operation	3.61 ± 0.82*	3.01 ± 0.63*	0.562	0.793
	*t* = 3.636, *P* < 0.05	*t* = 3.586 *P* < 0.05		
Last follow-up	8.31 ± 0.32*	7.75 ± 0.90*	0.592	0.771
	*t* = 4.543, *P* < 0.05	*t* = 4.562, *P* < 0.05		
Clinical signs				
Preoperation	4.23 ± 1.01	4.21 ± 1.03	0.381	0.996
3 days after operation	5.42 ± 0.38*	5.07 ± 0.84*	0.672	0.702
	*t* = 3.072, *P* < 0.05	*t* = 3.062, *P* < 0.05		
Last follow-up	5.53 ± 0.47*	5.39 ± 0.53*	0.432	0.853
	*t* = 4.252, *P* < 0.05	*t* = 4.322, *P* < 0.05		
Daily activity limitation				
Preoperation	5.07 ± 1.98	5.05 ± 1.95	0.390	0.931
3 days after operation	8.47 ± 2.96*	8.04 ± 2.37*	0.424	0.858
	*t* = 3.241, *P* < 0.05	*t* = 3.211, *P* < 0.05		
Last follow-up	12.73 ± 1.25* *t* = 4.562, *P* < 0.05	12.02 ± 1.46* *t* = 4.886, *P* < 0.05	0.521	0.799
Bladder function				
Preoperation	− 4.59 ± 1.82	− 4.63 ± 1.21	0.411	0.864
3 days after operation	− 0.74 ± 0.06*	− 1.02 ± 0.42*	5.213	0.011*
	*t* = 4.901, *P* < 0.05	*t* = 3.021, *P* < 0.05		
Last follow-up	− 0.29 ± 0.02*	− 0.31 ± 0.05*	0.872	0.056
	*t* = 5.625, *P* < 0.05	*t* = 4.952, *P* < 0.05		

Values are mean ± SD

*Statistically significant

## Discussion

Eighty percent of thoracolumbar burst fractures occurred in T10-L2, and most of them were adult males with high energy injury. Burst fractures are caused by vertical compression of the spine. If the fracture involves the middle column, the fracture block of the middle column protrudes into the spinal canal, which is the characteristic change of burst fracture. However, although some burst fractures involve the central column, there is no displacement of the posterior edge of the vertebral body to the spinal canal on the lateral X-ray film. CT scan can find that the bone cortex of the posterior edge of the vertebral body is not connected, and the bone block is slightly displaced [14]. Therefore, the main difference between compression fracture and burst fracture is whether the central column is involved [15]. With the increase of vertical compression force, the vertebral body changes from a compression fracture to a burst fracture. Compression fracture and burst fracture are different stages of a spinal fracture. The fracture is unstable due to the involvement of the anterior and middle columns. The purpose of the operation is to reduce the pressure, correct the deformity, and restore the normal curvature of the spine. Indirect decompression can be used if the nerve stimulation is small. Traditional indirect decompression relies on posterior or anterior traction and uses the integrity of ligament to reduce the fracture block. However, due to the interference of fascia and muscle tissue, the reduction effect is not perfect. In this study, the technique of manual reduction and indirect decompression was used. Screws were placed through the longest muscle and multifidus muscle space approach to avoid large area stripping of posterior spinal muscles [16], reduce intraoperative bleeding, avoid postoperative chronic low back pain, and retain the integrity of the posterior spinal muscle [17, 18]. While maintaining the strength to the ventral side, the pedicle screw was propped apart to make the anterior longitudinal ligament stretch and tighten and restore the height of the anterior column of the spine. At the same time, the posterior extension, posterior longitudinal ligament extension and tension, and posterior fracture block reduction, which the anterior and posterior forces were taken can restore the vertebral body height more effectively. With the increase of intervertebral space, it is easier to achieve anatomical reduction. However, due to the better reduction of the upper vertebral endplate and the recovery of the spinal canal diameter, the VAS score, the anterior height of injured vertebra, the wedge angle of the injured vertebral body, and the encroachment ratio of the injured vertebral canal were significantly improved compared with those before the operative improvement. The long-term follow-up showed that the anterior height of vertebra would not be lost with the extension of postoperative time, which was related to the better reduction during the operation. According to JOA score, the recovery of the bladder function in 3 days after operation was better than that before the operative improvement, which was related to better recovery of the spinal canal diameter.

## Conclusion

Manual reduction and indirect decompression in the treatment of thoracolumbar burst fracture can effectively restore the height of the vertebral body, reduce the wedge angle of the vertebral body, better restore the sagittal diameter of the vertebral canal, and recover bladder function faster after the operation, while the operation time and intraoperative blood loss are equivalent to those of traditional operation.

However, the sample size of this study is still small, and the postoperative follow-up time is not long enough. In the future work, we will choose to include more research objects, do a good job in long-term follow-up, and will also carry out biomechanical research under laboratory conditions.

## Data Availability

Not applicable.

## Abbreviations

CT: Computed tomography
MRI: Magnetic resonance imaging
JOA: Japanese orthopedic association
VAS: Visual analog scale
